# Do Native Insects and Associated Fungi Limit Non-Native Woodwasp, *Sirex noctilio*, Survival in a Newly Invaded Environment?

**DOI:** 10.1371/journal.pone.0138516

**Published:** 2015-10-08

**Authors:** Laurel J. Haavik, Kevin J. Dodds, Jeremy D. Allison

**Affiliations:** 1 Canadian Forest Service, Great Lakes Forestry Centre, Sault Ste. Marie, Ontario, Canada; 2 USDA Forest Service, Forest Health Protection, Durham, New Hampshire, United States of America; Institute of Plant Physiology and Ecology, CHINA

## Abstract

*Sirex noctilio* F. (Hymenoptera: Siricidae) is an introduced pest of pines (*Pinus* spp.) in several countries in the Southern Hemisphere. Although *S*. *noctilio* is established in North America (first discovered in 2004), it has not been a destructive pest there so far, where forest communities more closely resemble those in its native Eurasian range—where it is not a pest. To investigate the influence of the existing community of associated insects (competitors + natural enemies) and fungi (vectored by insects) on *S*. *noctilio* survival in North America, we examined stage-specific mortality factors and their relative importance, generating life tables drawn from experimentally-manipulated and natural cohorts of *Sirex* spp. (mostly *S*. *noctilio*, but some native *S*. *nigricornis* F.). For both natural and experimentally-manipulated cohorts, factors which acted during the earliest *Sirex* life stages, most likely tree resistance and/or competition among fungal associates, were paramount in dictating woodwasp survival. Experimentally-manipulated life tables revealed that protection from the community of associates resulted in a significantly, and substantially larger (>15x) *S*. *noctilio* F1 generation than exposure to it. Seventy percent of generation mortality in the exposed cohort was due to tree resistance or unknown causes early in larval development, which could have included competition among other bark- or wood-inhabiting insects and/or their fungal associates. Only 46% of generation mortality in the protected cohort was due to tree resistance and/or unknown causes. Parasitoids, particularly endoparasitoids (*Ibalia* spp.), showed limited ability to control *S*. *noctilio*, and reduced the experimentally-established cohort by only 11%, and natural cohorts an average of 3.4%. The relative importance of tree resistance vs. competition with bark- and wood-borers in reducing *S*. *noctilio* survival remains unclear. Tree resistance and/or competition likely contribute more than natural enemies in maintaining the *S*. *noctilio* population in North America below damaging levels.

## Introduction

Non-native invasive species negatively impact biodiversity and ecosystem functions, and are commonplace in many ecosystems worldwide. A better understanding of factors that contribute to the success or failure of invaders is critical for developing strategies to manage them. Once an invader is established, having overcome the challenges of proliferation from an often small founding population, spread and population growth, coupled with host availability, then determine whether it will become a destructive pest in its new environment (e.g. [1]). The lack of a co-evolutionary history between an herbivorous invader, its host plant(s), and components of its new ecosystem may contribute to its unchecked population growth. For instance, natural controls, such as natural enemies [2] or plant resistance [3] may be absent.

In the past few decades, invasive bark- and wood-inhabiting insects have caused considerable economic and ecological impacts in invaded habitats. Asian longhorned beetle has been repeatedly established in the U.S., and has established in Europe, resulting in large eradication programs where large numbers of trees have been cut in an attempt to eliminate populations [4, 5]. Emerald ash borer, a specialist on ash, has continued to spread in North America, where it threatens the existence of ash species [6]. Goldspotted oak borer, native to Arizona, but invasive in California, has killed many oaks in southern California and continues to spread northward [7, 8]. Red turpentine beetle, an occasional secondary pest of pine in North America, was introduced to China, where it spread rapidly, and has been responsible for extensive pine mortality in the past few decades [9]. The brown spruce longhorned beetle has established in Nova Scotia, Canada [10], and has the potential to displace a closely-related native species [11]. The European woodwasp, *Sirex noctilio* F., has established (first discovered in 2004, [12, 13]), and spread in northeastern North America, although so far it has not proved to be an aggressive tree-killing pest [14, 15].

*Sirex noctilio* is a pest of conifers in many other areas where it has been introduced (Hurley et al. 2007), yet it is not a pest in its native habitat [16, 17]. Presumably, similarities between forests in North America and in the native range (Eurasia) of *S*. *noctilio*, such as heterogeneity in tree species composition and a rich community of associated insects (competitors and natural enemies), have prevented *S*. *noctilio* from becoming an important invasive pest in North America [14, 15]. Patchy distribution of host material may negatively affect *S*. *noctilio* dispersal. Landscapes in northeastern North America are highly heterogeneous, where large areas of uniform pine plantations are uncommon. Also, *Pinus sylvestris*, native to Europe and Asia, and a favored host of *S*. *noctilio*, is widely planted and considered naturalized in northeastern North America [18]. In contrast, areas of the Southern Hemisphere where *S*. *noctilio* has established and become an exotic tree-killing pest are comprised of homogenous landscapes of non-native pine (e.g. *Pinus radiata*) monoculture plantations that lack a co-evolutionary history with *S*. *noctilio* and the environment in which they were planted [19, 20]. These pine stands often go unmanaged during early stand development resulting in an abundant habitat easily exploited by *S*. *noctilio*. Existing outside the native range of pine insect communities, those areas also lack natural enemies and competitors of *S*. *noctilio*. The degree to which the native insect community and its fungal associates can and has limited *S*. *noctilio* survival in North America has not been well-studied.

*Sirex noctilio* has a complex and well-studied life history. Briefly, it typically undergoes one generation per year, although it can require two or more years to complete development in cold climates [16, 21]. In mid-summer, females drill through the bark of pines and into the wood to oviposit eggs along with a phytotoxic mucus (noctilisin, [22]) and an obligate symbiotic fungus (*Amylostereum areolatum*, [23]), both of which act synergistically to weaken tree resistance and render pines suitable hosts for developing larvae [24, 25]. Females assess the quality of each potential oviposition site and drill one to five chambers in a cluster, drilling more chambers and inserting more eggs if tree condition is favorable [26, 27]. Eggs hatch inside these chambers, and larvae feed on *A*. *areolatum*, using the fungus to externally digest xylem [28], while creating meandering tunnels through the wood [29]. Larvae complete a variable number of instars (6–12, [26]) prior to pupation in late spring, usually of the following year.

The most important natural enemy of *S*. *noctilio* is a parasitic nematode, followed by two parasitoids, each acting at different times during *S*. *noctilio* development. *Ibalia leucospoides ensiger* (Norton) (Hymenoptera: Ibaliidae) and *Rhyssa persuasoria* (L.) (Hymenoptera: Ichneumonidae) are ideal natural enemies for *S*. *noctilio* in North America, since they are two of the most widely distributed parasitoids of the Siricinae in North America [30]. *Ibalia* spp. are endoparasitic kionobionts that attack eggs and early-instar siricids, have a similar seasonal phenology as *Sirex* spp., and generally complete one generation per year in North America (reviewed in [30]) [21]. *Rhyssa* spp. are ectoparasitic idiobionts that attack late-instar siricids [16, 30]. *Rhyssa* spp. may undergo a short or a long life cycle, with potentially two generations per year, whereby adults attack late-instar *Sirex* spp. (most likely *S*. *noctilio*) in the fall, and emerge the following spring to attack the same generation of late-instar *Sirex* spp. still developing inside trees [16, 21]. In other words, *Sirex* spp. larvae are vulnerable to attack from *Rhyssa* spp. for a relatively longer period of time than they are vulnerable to attack from *Ibalia* spp. The parasitic nematode, *Deladenus* (= *Beddingia*) *siricidicola* Bedding, has been widely used in biological control programs with varying success from very little to near complete control through sterilization of female *S*. *noctilio*, largely due to variation in virulence among different *D*. *siricidicola* strains [20, 31]. *Deladanus siricidicola* is present in North America, and was likely introduced along with *S*. *noctilio*, yet it remains in the wasp’s body cavity and does not penetrate eggs, which suggests that it is not capable of effectively sterilizing female wasps in North America [21, 32].

Some evidence exists that the native community of associated insects, and the fungi that they vector, affects *S*. *noctilio* in North America. *Sirex noctilio* commonly (90% of the time) shared habitat (pine boles) with several other subcortical phytophagous species (bark beetles and other wood borers, including *S*. *nigricornis* F.) [33], and a wide array of coniferous-inhabiting species have been captured arriving at *S*. *noctilio*-infested trees [34]. *Sirex nigricornis*, native to North America, may at times co-habit the same pine trees as *S*. *noctilio*. The two species share parasitoids; compete with other wood borers for habitat; and attack stressed pines—though *S*. *nigricornis* appears to require host material in a more advanced weakened condition than *S*. *noctilio* [21, 33, 35–37]. Indirect competition among bark beetles and *S*. *noctilio* through respective obligate symbiotic fungi likely occurs in North American forests, and fungi associated with these bark beetles out-competed *Amylostereum* spp. in the laboratory [38]. *Ibalia* spp. (mostly *leucospoides ensiger*), are the most abundant natural enemies recovered from *Sirex*-infested pine in North America, with reports of 10–20% parasitism, although *Rhyssa* spp. were also commonly collected [21, 35, 36, 39].

We investigated the influence of the existing community of insects, and the fungi that they vector, in recently-invaded pine forests on *S*. *noctilio* survival with two goals in mind. First, our results would indicate the importance of associates in limiting *S*. *noctilio* population growth in northeastern North America, which will aid in predictions of its behavior as it spreads further into eastern North America. And second, it may provide insight into characteristics of competitors or natural enemies in native communities with the capacity to mitigate impacts of potentially destructive non-native pests. Considerable attention has been paid to species that have become pests in their introduced environments (e.g.[40, 41]) compared with species that have not caused excessive damage. This study took advantage of the fact that *S*. *noctilio* has had little impact on North American pine forests to investigate the resistance of the native, existing insect and fungal community to a new invader.

Our objective was to determine whether the existing community of associates can or already does have a negative impact on *S*. *noctilio* survival, and to quantify the importance of specific mortality factors throughout woodwasp development. We employed a life table approach to examine factors involved in life stage-specific mortality throughout one year (one generation) in the woodwasp’s co-evolved host, *P*. *sylvestris*. We (1) examined the natural population of *Sirex* spp., sampling from cohorts at several sites throughout Ontario, and (2) experimentally manipulated *S*. *noctilio* cohorts that were exposed to, or protected from the natural community of associates. We predicted that *Sirex* mortality would be greatest in the earliest life stages, as is true of most other endophytic insects [42], probably due to tree resistance, which may prevent *A*. *areolatum* from establishing and creating a suitable habitat for larval development. Also, we expected that native parasitoids would limit *S*. *noctilio* survival.

## Methods

### Field methods: experimentally-manipulated cohorts

We selected an environment in which to establish manipulated *S*. *noctilio* cohorts that was representative of pine forests in Ontario with limited management. It was an unthinned, mixed *Pinus sylvestris*/*P*. *resinosa* plantation, near Angus, Ontario, where *S*. *noctilio* was present, but not in high densities (0.4 m^2^ha^-1^ of pine basal area attacked by *S*. *noctilio* in 2007 [14]). We obtained permission to conduct this study On the Brentwood tract from Simcoe County Forests. We felled 20 healthy, but small *P*. *sylvestris* (7–11 cm in diameter at breast height, dbh, and > 10 m tall) over a three-week period in July 2013. We cut a 3–4 m section from the mid-bole of each pine and placed each end of these logs onto a cinder block (25x10x10 cm). On the same day, we then secured two wire mesh (2x2 mm) screen cages (1 m long) to each horizontal log with heavy duty, plastic zip-ties at each end. The cages were equipped with Velcro® closures, sewn directly onto the wire mesh. To prevent cages from collapsing in on themselves, we screwed three wooden struts (10–15 cm long, cut from 5x5 cm lumber) into logs, equidistant apart (e.g. at 0°, 120°, and 240°), near both ends of cages, and encircled the struts in a hoop of plastic tubing (1.75 cm in diameter), screwed to the struts.

To artificially infest manipulated cohorts with *S*. *noctilio*, we obtained adults from infested pines in Innisfil, Ontario, identified by characteristic resin beading (resinosis) on the main bole [43]. These pines were cut in late June 2013, transported to the laboratory, and placed in large outdoor tents for collection of emerging adults. Each cage received two male/female pairs of *S*. *noctilio*, 1.5–3 weeks after pines were cut and caged, in an attempt to create a physically suitable (i.e. stressed, weakened) host tree for optimal survival of the F1 *Sirex* cohort (see [44]). Resin beading in response to *S*. *noctilio* attack was visible on most logs. To create the exposed cohort (n = 20), we removed one cage from each log (top or bottom section decided randomly by a coin flip) 1–2 weeks after wasps were inserted into cages. At this time all females were dead, and had presumably oviposited on logs. The remaining cages comprised the protected cohort (n = 20), and were left on logs throughout *Sirex* F1 development (or until October, see *Sampling different life stages* below).

### Field methods: natural cohorts

Because *Sirex*-infested pines were difficult to locate throughout much of Ontario, and because sites used in this study were also used to conduct other *Sirex*-related studies, we were only able to remove one *Sirex*-infested tree per sampling date (see *Sampling different life stages* below) at each site. We selected six sites, representing a range of *S*. *noctilio* activity levels (Table 1): *S*. *noctilio* was clearly dominant, both *S*. *noctilio* and *S*. *nigricornis* were common, or *S*. *noctilio* was newly established (first detected in 2013) and not yet dominant. It was not possible to determine whether a *Sirex*-attacked pine was attacked by *S*. *noctilio* or *S*. *nigricornis* until adults emerged from it. We identified *Sirex*-infested *P*. *sylvestris* as described above [43]. We obtained permission to removed trees from these sites from five private landowners, and the York Region Forests. We felled trees, cut the infested boles into ~0.5 m sections, and transported them to the laboratory to sample the different life stages of *Sirex*.

**Table 1:** *Sirex* spp. and parasitoid information at six sites throughout Ontario, from which natural life table cohorts were created.

Site/cohort	Dominant *Sirex* spp.	Density of *Sirex*-attacked pine in 2013 (trees ha^-1^)	Basal area of *Sirex*-attacked pine in 2013 (m^2^ ha^-1^)	*Sirex* within-tree density (per m^3^ wood)	*Ibalia* spp. (%)	*Rhyssa* spp. (%)
Innisfil	*S*. *noctilio*	390	3.84	584	17	2
Thames	*S*. *noctilio*	152	5.11	452	3	1
Little Lake	*S*. *noctilio*	87	1.16	286	35	0
Zephyr	both	43	1.25	280	5	12
Iron Bridge	*S*. *nigricornis*	87	1.11	68	0	0
Patton	*S*. *nigricornis*	65	2.95	10	0	0

Estimates of *Sirex* within-tree density and parasitism are based on the number of adult wasps that emerged from 2–10 infested pines cut in 2013 or 2014.

### Sampling different life stages

To estimate the number of *Sirex* in each life stage and quantify mortality factors throughout *Sirex* development, we cut, collected, and destructively sampled infested trees (natural cohorts) and logs (experimentally-manipulated cohorts) in October 2013 and again in June 2014 (Table 2). We collected trees and logs in late June, just prior to adult *S*. *noctilio* emergence, to provide *Rhyssa* spp. a maximal amount of time to attack *Sirex* larvae.

**Table 2:** Number of logs and different trees (*Pinus sylvestris*) collected for *Sirex* life tables and the actual number of surviving insects recovered from each life stage.

Treatment or site	No. logs (trees) Oct.	Volume wood Oct. (m^3^)	Total no. eggs/ neonates	Total no. small larvae	Total no. mid-sized larvae	No. logs (trees) Jun.	Volume wood Jun. (m^3^)	Total no. adults
E-Protected	9 (9)	0.07	1233	693	670	9 (9)	0.07	296
E-Exposed	9 (9)	0.06	1373	504	267	9 (9)	0.07	19
N-Innisfil	6 (1)	0.008	93	35	30	8 (1)	0.02	26
N-Thames	20 (1)	0.21	3124	1652	122	13 (1)	0.09	55
N-Little Lake	11 (1)	0.06	501	281	249	5 (1)	0.005	8
N-Zephyr	6 (1)	0.006	62	8	7	10 (1)	0.15	8
N-Iron Bridge	7 (1)	0.01	87	5	4	6 (1)	0.02	0
N-Patton	13 (1)	0.07	286	0	0	0	0	0

E and N refer to experimentally-manipulated and natural cohorts, respectively.

From infested material collected in October, we quantified the number of *Sirex* surviving and mortality factors that acted in egg/neonate, small-, and mid-sized larval life stages. We first peeled the bark from logs with a drawknife to reveal the sapwood surface. Then, to estimate egg/neonate densities, we used a dissecting microscope (6.4x), positioned over the log surface, to count the number and type (single, double, triple, etc.) of oviposition drills. Many oviposition drills were flooded with resin. We used an equation developed by Madden [27] to estimate the number of eggs oviposited by females (i.e. realized fecundity) based on drill type (Total no. eggs = 0.04*no. single drills + 0.68*no. double drills + 1.55*no. triple drills + 2.22*no. quadruple drills). We conducted a small test of this equation, by exposing 10 male/female *S*. *noctilio* pairs to a small (6–10 cm in diameter, 20–30 cm long) recently cut (~1 week prior) *P*. *sylvestris* log for one week, or until females died. We then dissected all logs by removing the bark with a drawknife and carefully shaving away layers of sapwood at each oviposition drill in search of eggs, also noting the type of drill. We dissected 467 oviposition drills. Our results were consistent with those of Madden (1974) (Total no. eggs = 0.03*no. single drills + 0.63*no. double drills + 1.51*no. triple drills + 2.28*no. quadruple drills).

To count the number of developing *Sirex* larvae, identifiable by a characteristic spine protruding from the terminal abdominal segment [45], we used a hammer and chisel to carefully pull layers of wood away beneath oviposition drills, and followed frass-filled galleries, which revealed larvae at various depths in the wood. We assumed that early instars (neonates) would not be visible via this sampling technique (without magnification), so estimates of the first life stage encompassed both the egg and early-instar (neonate) stages.

Larvae were either dead (yellow in color and deflated, and mortality was assumed to be tree- related—either resistance or nutrition) or alive and apparently healthy. We dissected all live larvae in search of endoparasitoids (most likely *Ibalia* spp.). Ectoparasitoids were also found, and we reared a subsample (~10) to the adult stage, all of which were *Rhyssa* spp. Some larvae were damaged during log dissections and could not be dissected. We estimated the number of damaged larvae presumed to be harboring an endoparasitoid based on the level of parasitism observed from the remaining intact, live larvae recovered from the same log sample. The total number of wasp larvae (ectoparasitoids + dead *Sirex* larvae + live, apparently-healthy *Sirex* larvae) recovered was the number of *Sirex* entering the small larval stage, and the number of healthy, un-parasitized larvae was the number of *Sirex* entering the mid-sized larval stage.

Logs and trees collected in late June 2014 were placed in cardboard rearing tubes located in a covered outdoor shed in Angus, Ontario (see [33] for a description). Rearing tubes were checked 5x per week, and adult *Sirex* and ectoparastoids were collected and counted from early July until early October, 2014. Competitors (bark beetles and cerambycid wood-borers) may emerge from and/or colonize pines prior to *Sirex*, so we were not able to quantify their direct or indirect effects on *Sirex* survival during development, or differentiate their effects from those of tree resistance. This mortality was categorized as “unknown” in life tables. Unknown mortality during the egg/neonate and small larval stages was likely due to tree resistance and/or competition, whereas unknown mortality during the mid-larval stage was likely due to overwintering. In the October log and tree collection, five of nine (55%) logs from the exposed cohort and two of the five trees from natural cohorts contained evidence of bark beetles and/or wood borers. Also, we were not able to identify whether *Sirex* were *S*. *noctilio* or *S*. *nigricornis* until the adult stage; when we were unsure, we referred to them as *Sirex*.

### Data analyses

Using the estimated numbers of *Sirex* entering each life stage (egg/neonate, small larval, mid-sized larval, and adult), we constructed life tables, and expressed wasp densities as numbers per unit volume of wood, so that comparisons could be made among all cohorts (see Table 2 for actual numbers). The notation that we used for life tables was as follows: x = life stage (October log/tree collection included egg—mid-larval stages; June included adult stage); n = volume of pine wood sampled; lx = number entering life stage, estimated per m^3^ of wood; dFx = mortality factor; dx = number dying during life stage.

All data analyses were conducted in R, version 3.1.1 [46]. We used generalized linear models (function = glm) to compare survivorship and mortality among and within life tables, fitted with appropriate error distributions. For all post-hoc testing, we used Tukey’s HSD in package “multcomp” [47]. Significant differences were determined from the z-statistic. Statistical significance was set at *P* < 0.05. To compare survivorship (no. of *Sirex* entering each life stage) between the two experimentally-manipulated cohorts, and with log as the experimental unit, we tested exposure to the associate community (exposed vs. protected) with a glm, and fit a poisson error distribution, because the response data were counts, with an added term in the model to account for overdispersion in the data (family = quasipoisson). Statistical comparisons in survivorship among sites were not made for natural cohorts because so few trees were collected from each site. To search for a relationship between *Sirex* density and mortality (i.e. the possibility of density dependent mortality), we employed Pearson’s correlations, with log as the experimental unit for experimentally-manipulated cohorts and site as the experimental unit for natural cohorts. For natural cohorts and with site as the experimental unit, we also used Pearson’s correlations to search for relationships between stage-specific mortality and site-level *Sirex* activity and parasitism, as well as the relationship between estimated small larval and parasitoid densities from life tables. Patton was excluded from analyses because mortality was 100% in the egg/neonate stage.

We examined both real and apparent mortality. Real mortality is a measure of the mortality incurred during a particular life stage relative to the number of individuals entering the life table (no. of eggs/neonates), and is used to determine, within a life table, in which life stage mortality is greatest [48]. In contrast, apparent mortality is mortality in a particular life stage relative to the number of individuals entering that life stage, and is useful for comparing relative levels of mortality among life tables, for a particular life stage [48]. To compare apparent mortality among cohorts, but separately for each life stage, and real mortality among life stages, but separately for each cohort, we used glm (family = binomial (logit)). For experimental cohorts, the number of “successes” (no. of *Sirex* that died) relative to the total number of trials (no. of *Sirex* entering a life stage or the life table for apparent or real mortality, respectively) was the true number, and for natural cohorts it was an estimate, adjusted for the volume of wood sampled. This was necessary for natural cohorts, because a different volume of wood was sampled among sites and at each site in October compared with June (range = 0.006–0.21 m^3^ of wood), whereas the total volume of wood that was sampled for each experimental cohort was similar between the two cohorts and log collection dates (0.06–0.07 m^3^).

For the experimentally-exposed cohort, we explored the relationship between *S*. *noctilio* larval density and total parasitoid (ecto- + endoparasitoids) as well as endoparasitoid density, first with a linear model (function = lm) and then with a polynomial model (function = lm(poly)), which provided a better fit (i.e. higher adjusted-R^2^). We examined residual and normal probability plots for violation of linear model assumptions (function = plot); no violations were detected.

## Results

### Life tables

**Fig 1:**
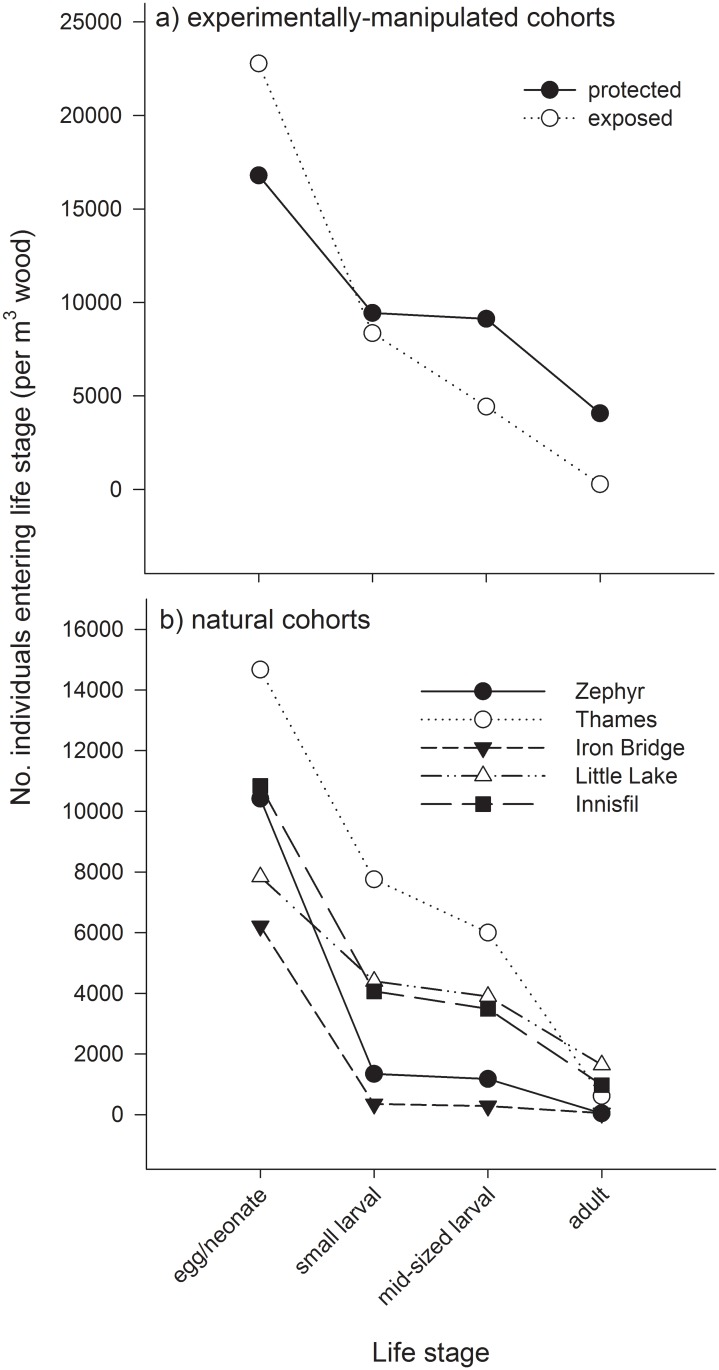
Survivorship curves from (a) experimentally-manipulated and (b) natural *Sirex* cohorts. Different symbols correspond to different (a) treatments or (b) sites, and represent an estimate of the total population density of *Sirex* recovered from (a) 9 logs (0.07 m^3^ of wood) or (b) one tree (0.006–0.21 m^3^ of wood each), which were/was collected in October (egg—mid-sized larval stages) or in June (adult stage).

In the experimentally-manipulated life tables (Table 3), the majority of mortality occurred during the egg/neonate stage (44% and 63% for the protected and exposed cohorts, respectively; real = apparent mortality for first life stage). Unknown mortality during the mid-larval stage (likely overwintering mortality) was also very high, accounting for 30% and 17% of real mortality in protected and exposed cohorts, respectively. In the exposed cohort, endo- and ectoparasitoids accounted for 9% and 2% of real mortality, during development (egg—mid-larval). In the exposed cohort, endo- and ectoparasitoids accounted for 24% and 4% of apparent mortality, in the small larval stage, and ectoparasitoids accounted for 8% of apparent mortality in the mid-sized larval stage. The protected cohort was slightly smaller (about three-quarters as large) than the exposed cohort initially; yet it was more than 15 times greater by the time adults emerged (Table 3, Fig 1).

**Table 3:** Partial age-specific life tables (2013–2014), including mortality factors, from experimentally-established *Sirex noctilio* populations, which were protected from or exposed to the naturally-occurring community of associate insects and fungi in southern Ontario.

Life table	x	n (m^3^ wood)	lx	dFx	dx	Apparent mortality (%)	Real mortality (%)	Generation mortality (%)
Protected	egg/neonate	0.07	16796	unknown	7354	44	44	
	small larval	0.07	9442	tree-related	313	3	2	
				endoparasitoid	0	0	0	
				ectoparasitoid	0	0	0	
				unknown	1	0	0	
				total	314	3	2	
	mid-sized larval	0.07	9128	ectoparasitoid	0	0	0	
				unknown	5057	55	30	
				total	5057	55	30	
	adult	0.07	4071					76
Exposed	egg/neonate	0.06	22781	unknown	14419	63	63	
	small larval	0.06	8362	tree-related	1211	14	5	
				endoparasitoid	2046	24	9	
				ectoparasitoid	315	4	1	
				unknown	360	4	2	
				total	3932	47	17	
	mid-sized larval	0.06	4430	ectoparasitoid	334	8	1	
				unknown	3820	86	17	
				total	4154	94	18	
	adult	0.07	276					99

x = life stage; n = volume of pine wood sampled: 9 logs from different trees for each population and collection (fall collection included egg—mid-larval stages; spring included adult stage); lx = number entering life stage, estimated per m^3^ of wood; dFx = mortality factor; dx = number dying during life stage.

Only *S*. *noctilio*, no *S*. *nigricornis*, adults emerged from natural cohorts. Similar to the experimentally-manipulated life tables, mortality in the natural life tables was greatest during the egg/neonate stage (44–100%; Table 4). Mortality during this earliest life stage was especially high at sites where *S*. *nigricornis* was the dominant species and *S*. *noctilio* was new (Iron Bridge = 94%, Patton = 100%) or both species were present (Zephyr = 87%) (Table 4). Real mortality due to unknown factors in the mid-sized larval stage, which was most likely due to overwintering, was also high, especially among cohorts that experienced relatively less mortality during the egg/neonate stage (range among Innisfil, Thames, and Little Lake = 23–37%). Among natural *Sirex* life tables, endo- and ectoparasitoids accounted for very little, 0–6% and 0–2%, of real mortality during development. Within the small larval stage, endo- and ectoparasitoids accounted for 0–14% and 0–4% of apparent mortality, which was much less than in the experimentally-exposed life table. At Thames, ectoparasitoids accounted for < 1% of apparent mortality during the mid-larval stage. No ectoparasitoids were recovered from the mid-larval stage in any of the other natural cohorts.

**Table 4:** Partial age-specific life tables (2013–2014), including mortality factors, from the natural *Sirex* population at six sites throughout Ontario.

Life table Dominant species	x	n (m^3^ wood)	lx	dFx	dx	Apparent mortality (%)	Real mortality (%)	Generation mortality (%)
Innisfil	egg/neonate	0.01	10841	unknown	6774	62	62	
*S*. *noctilio*	sm. larval	0.01	4067	tree-related	0	0	0	
				endoparasitoid	581	14	5	
				ectoparasitoid	0	0	0	
				unknown	0	0	0	
				total	581	14	5	
	mid. larval	0.01	3486	ectoparasitoid	0	0	0	
				unknown	2514	72	23	
				total	2514	72	23	
	adult	0.02	972					91
Thames	egg/neonate	0.21	14669	unknown	6912	47	47	
*S*. *noctilio*	sm. larval	0.21	7757	tree-related	620	8	4	
				endoparasitoid	836	11	6	
				ectoparasitoid	300	4	2	
				unknown	0	0	0	
				total	1756	23	12	
	mid. larval	0.21	6001	ectoparasitoid	12	0	0	
				unknown	5374	90	37	
				total	5386	90	37	
	adult	0.09	615					96
Little Lake	egg/neonate	0.06	7837	unknown	3443	44	44	
*S*. *noctilio*	sm. larval	0.06	4394	tree-related	203	5	3	
				endoparasitoid	297	7	4	
				ectoparasitoid	0	0	0	
				unknown	0	0	0	
				total	500	11	6	
	mid. larval	0.06	3894	ectoparasitoid	0	0	0	
				unknown	2254	58	29	
				total	2254	58	29	
	adult	0.005	1640					79
Zephyr	egg/neonate	0.006	10416	unknown	9069	87	87	
both	sm. larval	0.006	1347	tree-related	168	12	2	
				endoparasitoid	0	0	0	
				ectoparasitoid	0	0	0	
				unknown	1	0	0	
				total	169	13	2	
	mid. larval	0.006	1178	ectoparasitoid	0	0	0	
				unknown	1131	96	11	
				total	1131	96	11	
	adult	0.150	47					99
Iron Bridge	egg/neonate	0.01	6217	unknown	5866	94	94	
*S*. *nigricornis*	sm. larval	0.01	351	tree-related	70	20	1	
				endoparasitoid	0	0	0	
				ectoparasitoid	0	0	0	
				unknown	1	0	0	
				total	71	20	1	
	mid. larval	0.01	280	ectoparasitoid	0	0	0	
				unknown	232	83	4	
				total	232	83	4	
	adult	0.02	48					99
Patton	egg/neonate	0.07	4235	unknown	4235	100	100	
*S*. *nigricornis*	sm. larval	0.07	0					100

x = life stage; n = volume of pine wood sampled: one tree for each collection (fall collection included egg—mid-larval stages; spring included adult stage); lx = number entering life stage, estimated per m^3^ of wood; dFx = mortality factor; dx = number dying during life stage.

### Survivorship

**Fig 2:**
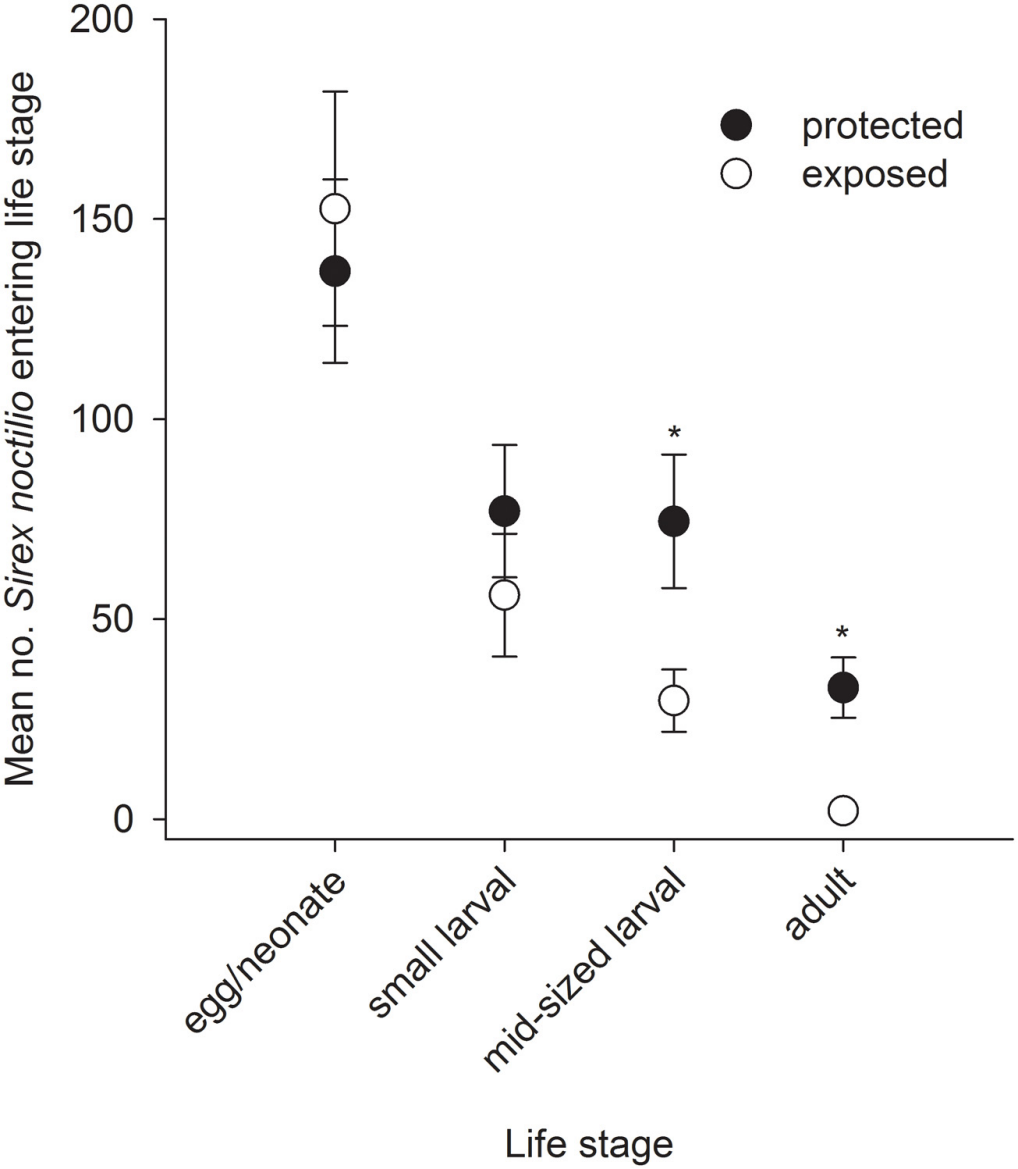
Mean number of *Sirex noctilio* at the beginning of each life stage in experimentally-manipulated cohorts. For each treatment, dots represent the mean number of *S*. *noctilio* recovered from 9 logs (~0.07 m^3^ wood) that were collected in October (egg/neonate—mid-sized larval), or from a second set of 9 logs (0.07 m^3^ wood) that were collected in June (adult), and error bars represent standard errors. Cohorts were either protected from (filled dots) or exposed to (open dots) the naturally-occurring community of associates. The effect of exposure to associates was tested separately for each life stage. *P < 0.01 according to Tukey’s HSD.

All life tables exhibited a Type IV survivorship curve (according to Slobodkin 1962), wherein mortality acted more severely on the earliest life stage, as evidenced by the steepest portion of the curves existing between the first two life stages (Fig 1). Densities of surviving *S*. *noctilio* were not different between the experimentally-manipulated cohorts in the first two life stages, but they were significantly greater in the protected compared with the exposed cohort in the final two life stages (z = 2.49; df = 1,16; *P* = 0.012 and z = 3.64; df = 1,16; *P* < 0.001 for mid-sized larval and adult stages, respectively; Fig 2). The number of mid-sized larvae and adults were, on average, 2.5 and 16.5 times larger in the protected compared with the exposed cohort. At most sites, the shape of survivorship curves for natural cohorts (Fig 1b) was similar to that of the experimentally-protected cohort (Fig 1a); Thames was most similar to the experimentally-exposed cohort.

### Mortality

Apparent mortality was significantly greater in the experimentally-exposed than in the protected cohort during all life stages (z = 9.91; df = 1; *P* < 0.001, z = 14.14; df = 1; *P* <0.001, and z = 9.32; df = 1; *P* <0.001 for egg/neonate, small larval, and mid-sized larval stages, respectively, Table 5). Apparent mortality in the small larval stage was more than 15 times greater in the experimentally-exposed (47%) than in the protected cohort (3%). In natural cohorts (Table 5), there was more variability among sites in apparent mortality during the egg/neonate than during the small- and mid-larval stages (44–100% vs. 11–23% and 58–96%, for egg/neonate vs. the small- and mid-sized larval stages, respectively).

**Table 5:** Differences in apparent mortality (%) among life tables of *Sirex* developing in *Pinus sylvestris* by treatment for experimentally-manipulated cohorts and separately by site for natural cohorts.

Life stage	Protected	Exposed	Innisfil	Thames	Little Lake	Zephyr	Iron Bridge
Egg/neonate	44 b	63 a	62 B	47 C	44 C	87 A	94 A
Small larval	3 b	47 a	14 AB	23 A	11 B	13 AB	20 AB
Mid-sized larval	56 b	93 a	72 AB	90 A	58 B	96 AB	83 AB

Each life stage was tested separately using a generalized linear model. Different letters indicate statistically significant differences (*P* < 0.05) among cohorts according to Tukey’s HSD. Lowercase letters indicate differences between experimentally-manipulated cohorts, and uppercase letters indicate differences among natural cohorts (sites).

Comparisons of real mortality by life stage revealed that the egg/neonate stage incurred significantly more mortality than all other life stages, which was a consistent pattern for all life tables (44–100%; Table 6). Real mortality during the two larval stages was not different in the exposed cohort (Table 6), but was greater in the mid-larval than the small larval stage in the protected cohort (30% vs. 2%; Table 6). Similar to the protected cohort, real mortality during the mid-sized larval stage in natural cohorts was intermediate (4–37%), and lowest during the small larval stage (1–12%; Table 6).

**Table 6:** Differences in real mortality (%) among life stages of *Sirex* developing in *Pinus sylvestris*, tested separately for treatments (experimentally-manipulated cohorts) or sites (natural cohorts).

Treatment/site	Egg/neonate	Small larval	Mid-sized larval
Protected	44 a	2 c	30 b
Exposed	63 a	17 b	18 b
Innisfil	62 a	5 c	23 b
Thames	47 a	12 c	37 b
Little Lake	44 a	6 c	29 b
Zephyr	87 a	2 c	11 b
Iron Bridge	94 a	1 c	4 b

Different letters indicate statistically significant differences (*P* < 0.05) among life stages according to Tukey’s HSD, tested separately for each row, using generalized linear models.

### Density relationships

There was no evidence for density dependent mortality in either the experimentally-manipulated cohorts or in the natural cohorts. For the natural cohorts, comparisons between site-level *Sirex* activity (current and cumulative) and parasitism (Table 1), and *Sirex* density and apparent mortality at different life stages, revealed that density of eggs/neonates was significantly positively correlated with cumulative basal area of pine attacked by *Sirex* at a site (r = 0.857; *P* = 0.029, not shown), and apparent mortality in the small larval stage was negatively correlated with mean percentage of *Ibalia* parasitism at a site (r = -0.903; *P* = 0.036, not shown). From natural cohorts, estimated densities of small larvae were significantly, positively correlated with estimated densities of total parasitoids (r = 0.950; *P* = 0.004, not shown) and endoparasitoids (r = 0.948; *P* = 0.004, not shown), but not ectoparasitoids. All other pairwise correlations were not significant.

**Fig 3:**
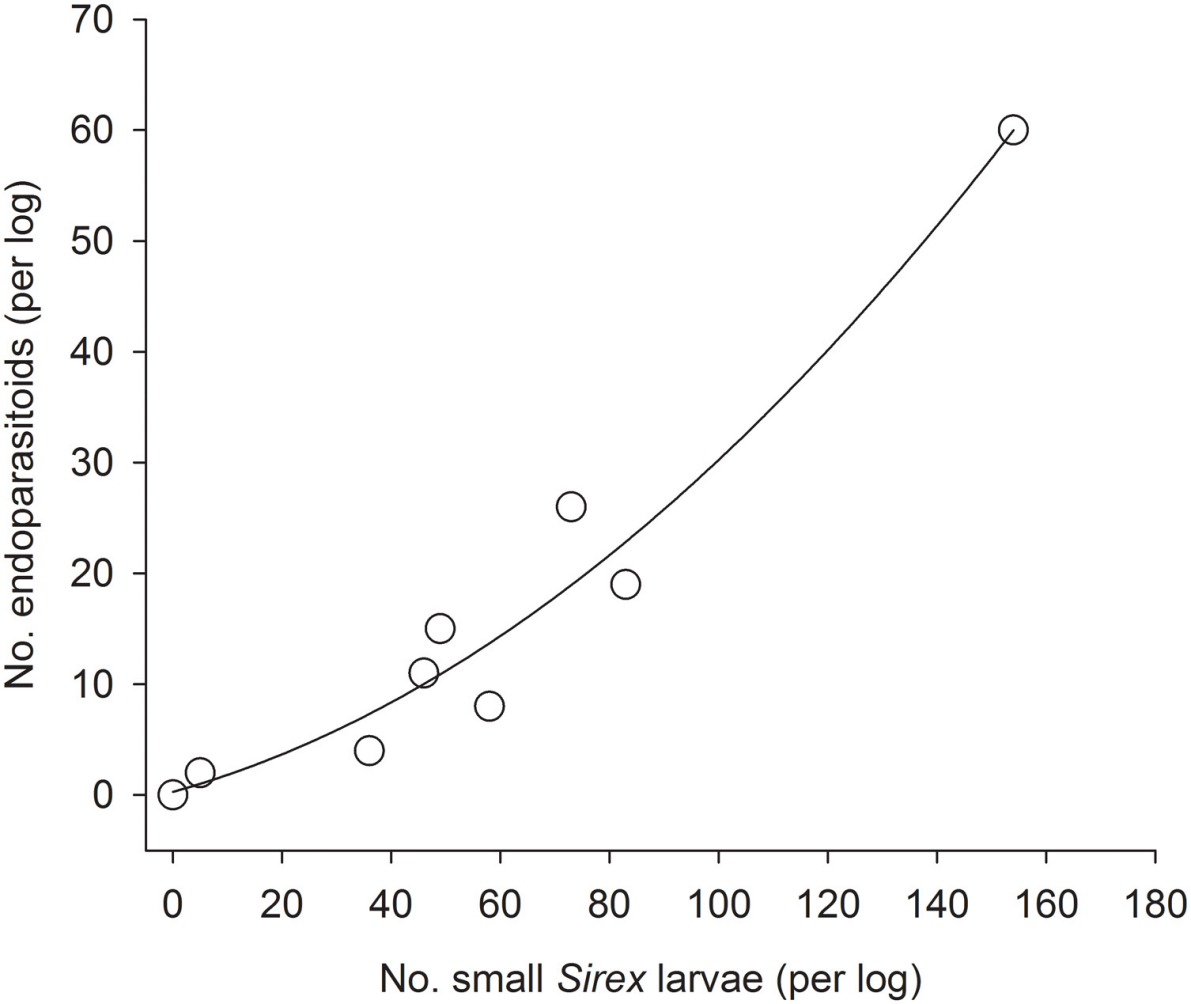
Relationship between number of small *Sirex* larvae and endoparasitoids from the experimentally-manipulated, exposed cohort. Equation of the fitted line: y = 0.2906 + 0.1365x + 0.0016x^2^; adjusted R^2^ = 0.94; *P* < 0.001. Each dot represents number of wasps recovered from one log.

Endoparasitiods in the experimentally-exposed cohort displayed a Type III functional response (Fig 3). As the number of small *Sirex* larvae per log increased, so did the number of *Sirex* that were parasitized, and at an accelerating rate (y = 0.2906 + 0.1365x + 0.0016x^2^; *F* = 62; df = 2,6; *P* < 0.0001; adjusted R^2^ = 0.94). High enough densities of *Sirex* to induce a saturation point (i.e. a sigmoidal curve with a clear upper asymptote) were not present in our study. Number of all parasitioids (endo- + ectoparasitoids) exhibited a similar significant response to number of small *Sirex* larvae (y = 1.0478 + 0.0798x + 0.0025x^2^; *F* = 80; df = 2,6; *P* < 0.0001; adjusted R^2^ = 0.95, not shown). Ectoparasitoids were only recovered from four logs, and so were not analyzed separately.

## Discussion

### Community resistance: trees

High levels of early-stage mortality indicate that initial conditions in pines were very important for *Sirex* survival. This same trend was reported from life tables of other wood-inhabiting insects [49–52]. It follows that mechanisms of tree resistance would be most effective at the early stages of insect attack: female oviposition or larval establishment. Indeed, tree resistance appears to be the most important factor limiting populations of many bark- and wood-boring insects (e.g. [52–56]).

Tree resistance is clearly an important obstacle for *Sirex* to overcome. The combined action of a symbiotic fungus and phytotoxic mucus represent a co-evolved mechanism for *Sirex* to combat a well-defended host habitat. *Sirex noctilio* oviposition drills are often flooded with resin when a tree is unsuccessfully attacked, killing eggs and small larvae [57–59]. We observed this constitutive resistance in many of the trees that we sampled—even on pine logs two weeks after cutting. In fact, all oviposition drills (~2900 eggs, in ~1 m^3^ of wood) were flooded with resin in our first attempt at this study in 2012, when we conditioned pines by girdling two weeks prior to infestation with *S*. *noctilio*. Pines also produce polyphenols in response to *S*. *noctilio* attack, in the sapwood surrounding the attack site [60, 61]. This induced defensive response contains a high amount of pinosylvin, a fungicidal compound that likely prevents *Amylostereum* spp. from establishing [61]. Female *S*. *noctilio* will condition pines prior to oviposition, by injecting noctilisin and *A*. *areolatum* only, rendering trees susceptible to successful attack (i.e. larval development) in succeeding years [58]. In North America, *S*. *noctilio* (and *S*. *nigricornis*) usually kill, and develop within, unhealthy or suppressed pines [14]–those trees that are likely to have compromised constitutive and induced resistance capacity.

### Community resistance: competitors

We were not able to separate the negative effects of tree resistance or overwintering from those of competitors (direct or indirect) on *Sirex* survival. Interspecific competition could have been important, given that apparent mortality was significantly higher in the experimentally-exposed compared with the protected cohort during all life stages (Table 5), and real mortality not attributable to parasitism was very high (76–100%) in natural cohorts, although some of that mortality was undoubtedly due to tree resistance and overwintering. Bark-beetle vectored fungi, *Leptographium wingfieldii* Morelet and *Ophiostoma minus* (Hedgcock) H. and P. Sydow, were more aggressive colonizers than *A*. *areolatum* of artificial media in the laboratory [38]. *Amylostereum areolatum* was not able to colonize media already occupied by bark-beetle fungi [38]. Bark beetles colonize pines earlier in the season than *S*. *noctilio* [33], which may afford their fungal associates an advantage over *A*. *areolatum*. General patterns among endophytic insects indicate that they suffer the most mortality from natural enemies and the least from competitors, with tree resistance being intermediate [42]. Our results indicate that natural enemies appear to be less important than other factors, such as tree resistance, and possibly competition, in limiting *S*. *noctilio* in North America.

### Community resistance: parasitoids

Parasitoids, particularly endoparasitoids (*Ibalia* spp.), demonstrated limited ability to control *S*. *noctilio*, and reduced the experimentally-exposed *S*. *noctilio* cohort by only 11%. However, real mortality during the small-larval stage was significantly, and substantially smaller in the protected (2%) compared with the exposed cohort (17%), indicating that parasitism, especially by *Ibalia*, limited *S*. *noctilio* survival. Interestingly, life tables constructed from the natural *Sirex* population were more similar to the protected cohort in terms of mortality; extremely little mortality occurred during the small-larval stage (1–12%), when *Ibalia* parasitism became evident. In our experimental setting, *Ibalia* had a measureable, albeit small, negative effect on *S*. *noctilio* survival; yet this was not the case in the natural population, where parasitism reduced *Sirex* survival by a mean of 3.4%.

Similar to our results, parasitism has been noted for other bark- and wood-boring insects ([53, 62],[55]; reviewed by [56]), including *S*. *noctilio* in North America [21, 35, 36, 39], but these studies indicated that parasitoids do not exert enough impact (≤ 50% parasitism) to control borer populations. There are many possible explanations for this. For example, parasitoids may be generalists, and thus not explicitly co-evolved with the biology of the specific subcortical insect (e.g. *Megarhyssa* spp.). But most importantly, whether a specialist or a generalist, and for whatever reason, parasitoids may not have the ability to respond functionally or numerically to increases in their host populations.

The life history of *Ibalia* spp. and *Rhyssa* spp., and their efficacy as biological control agents in other areas, helps to interpret why they had such a low impact on *Sirex* in our study. Life history traits of *I*. *leucospoides* have facilitated its widespread establishment in areas where it has been introduced as a biological control agent (see Table 3, [63]). For example, *I*. *leucospoides* is phenologically synchronized with *S*. *noctilio*; it has a high potential fecundity (~600 eggs, 75% of which are mature upon adult emergence, [64]); it exhibits a functional response [65] and is a good forager; it can detect host patch richness from a distance and adjust foraging time accordingly [66, 67]. However, other traits limit the success of *I*. *leucospoides* in controlling *S*. *noctilio* populations. *Ibalia leucospoides* has a long handling time (5–20 minutes per oviposition), which results in a sigmoidal functional response with a saturation point [65, 66]. Local *Ibalia* spp. densities may be slow to respond to increasing *Sirex* densities due to their solitary lifestyle. Our observations from natural cohorts, that endoparasitism occurred at most sites, but at low frequency, were generally consistent with these ideas.

In our study, *Ibalia* spp. were likely more successful when confronted with larger initial densities of *Sirex*. The natural population of *S*. *noctilio* at the site we used to experimentally manipulate cohorts was low. Consequently, we expected that the parasitoid population was also low; yet, when presented with essentially a *Sirex*-larvae-buffet in our artificially-infested logs (mean = 56, maximum = 154 larvae per log), endoparasitoids exhibited a functional response, with higher numbers of parasitoids recovered from logs that also had higher numbers of *Sirex* larvae. We did not observe a saturation point, which suggests that the existing population of *Ibalia* spp., at least in that forest, was not limited by handling time. Among natural cohorts, sites with the largest densities of small larvae also had greater incidence of parasitism (Tables 1 and 4). Finally, endoparasitism could have been greater than what we reported, because we did not recover and dissect eggs and neonates. *Ibalia* spp. could have been responsible for some *Sirex* mortality during the egg/neonate stage that did not result in successful parasitoid development.

Ectoparasitoids, most likely *Rhyssa* spp., were substantially less common in our study than endoparasitoids. An intensive life table, constructed in Australia before *D*. *siricidicola* was introduced, provided long-term evidence (10 years) that *Rhyssines* (*M*. *nortoni* (Cresson) + *R*. *persuasoria* (L.)) were able to limit a *S*. *noctilio* population in a delayed density-dependent manner [68]. Taylor’s [68] study also determined that *R*. *persuasoria* emergence was greatest during the second spring, more than one year after pines were attacked by *S*. *noctilio*. In contrast, Ryan et al. [33] found that only a very small amount (≤ 5%) of the wasp population (*Sirex* + parasitoids) required more than one year to complete development in Ontario. Further, re-infestation of the same (natal) logs upon adult emergence could not likely be ruled out for either study. These differences could be due to environment; Taylor [68] used emergence cages to capture adult insects on live/dying pines, whereas we (and Ryan et al. [21, 33]) stored cut logs in a covered shed, which likely reduced insect development time through heating and drying of host material. Removal of *Sirex*-infested pines from Ontario forests in late spring, when at least a portion of the *Rhyssa* spp. population were actively seeking hosts, further complicates our results, and may have led to an underestimate of the impact of *Rhyssa* spp. on *S*. *noctilio* in North America.

### Predictions for *S*. *noctilio* in North American pine forests

The native ecology in North American pine forests, including the community of associated insects and fungi that they vector, and pine resistance, reduced *S*. *noctilio* survival. However, the relative importance of tree resistance vs. competition among bark- and wood-borers and their fungal associates remains unclear. Future research should focus on this as well as improving tree resistance, and understanding what factors prevent parasitoids from effectively controlling *S*. *noctilio*. For instance, silvicultural treatments can improve pine health and reduce the number of *S*. *noctilio*-attacked trees by 75% [69]. And, *Ibalia* spp. may be more successful in warmer climates, where *Sirex* diapause is short, and *Rhyssa* spp. may be more successful in colder climates, where *Sirex* diapause is long, allowing *Rhyssa* spp. more search time [16, 68, 70]. Such variability in parasitoid success may have different implications for pine ecosystems in the southeastern U.S. vs. those in Canada.

## Conclusions

Life table analyses indicated that factors which acted during the earliest *Sirex* life stages, most likely tree resistance and/or competition among fungal associates, were paramount in dictating woodwasp survival. Natural enemies, which included *Ibalia* spp. and *Rhyssa* spp., were not important mortality factors for *Sirex*, especially in cohorts drawn from the natural population. Experimentally-manipulated life tables revealed that protection from the community of associates resulted in a substantially larger (~15x) F1 generation than exposure to it. Seventy percent of generation mortality in the experimentally-exposed cohort was due to tree resistance (resin flooding the oviposition drills, and/or polyphenols that prevented *A*. *aereolatum* from establishing) or unknown causes early in larval development, which could have included competition among other subcortical insects and/or their fungal associates. Only 46% of generation mortality in the experimentally-protected cohort was due to tree resistance and/or unknown causes. In natural cohorts, generation mortality due to tree resistance, fungal competition, and/or unknown causes early in larval development was more variable, and ranged from 47 to 100% among sites.
